# ACT Values after Neutralization Lower than Pre-heparinization ACT Leads to Lower Operative Times, Bleeding, and Post-Operative Transfusions in CABG Patients: an Observational Study

**DOI:** 10.21470/1678-9741-2018-0116

**Published:** 2018

**Authors:** Weitie Wang, Yongwang Wang, Jinshan Wang, Rihao Xu, Junwu Chai, Wei Zhou, Honglei Chen, Fenlong Xue, Xiangrong Kong, Wang Kai

**Affiliations:** 1 Department of Cardiovascular Surgery, 1st Central Hospital of Tianjin, Tianjin, China.; 2 Department of Cardiovascular Surgery, 2nd Hospital of Bethune, Jilin University, Changchun, Jilin, China.; 3 Department of Anesthesiology, 1st Central Hospital of Tianjin, Tianjin, China.; 4 Department of Cell Transplantation, 1st Central Hospital of Tianjin, Tianjin, China.

**Keywords:** Cardiopulmonary Bypass, Coronary Artery Bypass, Thromboelastography, Whole Blood Coagulation Time, Protamines

## Abstract

**Objective:**

To evaluate if lower activated coagulation time (ACT) value after neutralization than preoperative ACT value was effective in reducing bleeding, operative times, and post-operative transfusions in patients underwent coronary artery bypass grafting (CABG).

**Methods:**

Retrospective selection of 398 patients from January 2014 to May 2017. Patients were divided into 2 groups according to final ACT after neutralization: A - final ACT lower than preoperative ACT; and B - final ACT higher than or equal to preoperative ACT. Hemostatic time, intraoperative blood loss, ACT after final neutralization, mediastinal blood loss, and transfusion requirements were observed.

**Results:**

The hourly blood loss in the Group A was generally lower than in the Group B at first 3 hours, which has significant difference (*P*<0.05). However, there was no difference after 3 hours between the two groups. Operative time, intraoperative blood loss, mediastinal blood loss, transfusion requirements, and drainage in the first postoperative 12 hours in the Group A were lower than in Group B, which has significant difference (*P*<0.05).

**Conclusion:**

As a result, final ACT values lower than pre-heparinization ACT values are safe and lead to lower operative times, bleeding, and post-operative transfusions.

**Table:** 

Abbreviations, acronyms & symbols	
**ACT**	**= Activated coagulation time**
**AKI**	**= Acute kidney injury**
**CABG**	**= Coronary artery bypass grafting**
**CPB**	**= Cardiopulmonary bypass**
**DSWI**	**= Deep sternal wound infection**
**ECG**	**= Electrocardiogram**
**HB**	**= Hemoglobin**
**ICU**	**= Intensive care unit**
**LMWH**	**= Low molecular weight heparin**
**PLT**	**= Platelet**
**TEG**	**= Thromboelastography**

## INTRODUCTION

Cardiopulmonary bypass (CPB) is a component of cardiac surgeries which requires full systemic heparinization before it is started^[1]^. Protamine is administered to neutralize the heparin's anticoagulant effect after CPB to return to normal hemostasis. Activated coagulation time (ACT) has been widely used to monitor the heparin's intraoperative anticoagulant effect, it needs to exceed 480 seconds before starting CPB and it is required to be equal to the preoperative value after neutralization. However, we found less bleeding in some cases whose final ACT after neutralization was a little lower than pre-heparin ACT. In these cases, the closing time was shorter than the pre-heparin one. Thereafter, a retrospective observational study was performed on patients to compare the influence of the final ACT on the outcomes of cardiac patients.

## METHODS

This is a retrospective observational study conducted from January 2014 to May 2017. All patients undergoing on-pump coronary artery bypass grafting (CABG) were enrolled. Exclusion criteria included patients: 1) with liver disease, 2) renal disease, 3) known bleeding diathesis, 4) who received antiplatelet (aspirin) or fibrinolytic agents within 5 days before surgery, 5) with platelet (PLT) count < 100*10^9^, 6) abnormal preoperative thromboelastography (TEG) parameters, 7) preoperative infections, 8) preoperative anemia, and 9) incomplete clinical records. Finally, 398 patients were enrolled in this study. This study was approved by the Clinical Trial Ethics Committee of the 1^st^ Central Hospital of Tianjin (Certificate no: E2016023L).

Patients were divided into 2 groups according to the final ACT after neutralization before they were transferred to intensive care unit (ICU). Patients whose final ACT was lower than preoperative ACT were in the Group A and those with final ACT equal to or higher than preoperative ACT were in the Group B. The following parameters were collected from our database: gender, height, weight, body surface area, CPB time, aortic cross-clamp time, minimum temperature reached during CPB, basal ACT, initial heparin bolus, post-heparinization ACT, ACT levels during CPB, final ACT, closing time, intraoperative blood loss, the first 12 hours and total bleeding from the chest drainage, need for transfusion in the ICU, and need for reoperation resulting from bleeding and other complications.

### Heparin and Protamine Management

Basal ACT was monitored initially after the patient was transferred to the operation room and it was used as a reference value after neutralization. At the same time, another blood sample was collected to determine the baseline coagulation ability by using heparin-modified TEG.

Heparin (Tianjin Biochemical Pharmaceutical Co Ltd, Tianjin, China) was given at an initial dose of 3 mg/kg to achieve an ACT (Medtronic Inc., Minneapolis, MN, USA) higher than 480 seconds before CPB, which was instituted with an ascending aortic cannula and a two-stage right atrial cannula in all patients. Moderate systemic hypothermia (28-30ºC) was maintained during CABG. ACT was measured every 30 minutes during CPB to monitor intraoperative heparin. Additional heparin bolus (5.000 U) was given if the ACT was < 480 seconds. After discontinuation of CPB, heparin was reversed with a bolus of protamine sulphate using the heparin-ACT dose-response curve method to determine the dosage of protamine. Actually, ACT was measured (30 minutes later) after the first neutralization. Additional small doses of protamine (10-25 mg) were administered in patients whose ACT was still higher than the pre-heparin values after the first neutralization and even if the ACT was between 140-200 according to the anesthesiologist's experience. Final blood samples were collected for ACT and TEG assays at the same time. The initial dose plus the additional doses of protamine are equal to the total dose of protamine in the surgery.

All residual post-CPB cardiotomy blood was transfused through the venous line before protamine administration. Cell-saving devices were used in all patients. Recollected blood and other blood products were transfused if hemoglobin (HB) was < 80 g/L. ACT was measured by using 2 mL of blood. TEG was carried out by a TEG Hemostasis Analyzer (Beijing Universal Medical Technology Co Ltd, Beijing, China); we measured basal ACT, post-heparinization, and final levels in normothermic conditions. All measurements were done according to the manufacturer's instructions.

All surgeries were performed by the same surgeons, anesthesiologists, and perfusionists. The anesthesiologist is the only one in charge of determining the dose of protamine.

### Clinical Outcomes

- Surgical mortality: death occurring in hospitalization.
- Re-sternotomy for bleeding: reoperation to control bleeding within 36 hours following initial surgery.
- Postoperative myocardial infarction: the appearance of new Q waves in 2 or more contiguous leads on the electrocardiogram (ECG).
- Atrial/ventricular arrhythmia after surgery: any episode of atrial/ventricular fibrillation that was registered by the monitoring system on a rhythm strip or the 12-lead ECG.
- Hemostatic time: time between the first protamine injection until sternal closure.
- Postoperative respiratory failure: duration of mechanical ventilation > 72 hours or re-intubation following surgery.
- Postoperative pneumonia: a positive result in a sputum culture requiring anti-infective treatment or the chest X-ray diagnosis of pneumonia following cardiac surgery.
- Stroke: new permanent neurological event.
- Deep sternal wound infection (DSWI): bone related, any drainage of purulent material from the sternotomy wound and instability of the sternum.
- Acute kidney injury (AKI): defined and classified according to the criteria proposed by the Acute Kidney Injury Network.
- Adverse events of protamine: were caused directly by protamine, occurred within 30 minutes after the initiation of protamine, and met one or more of the following criteria - (1) decrease in systemic arterial blood pressure; (2) increase in pulmonary arterial pressure of at least 25% resulting in decrease of systemic arterial blood pressure as defined in (1); (3) non-cardiogenic pulmonary edema; and (4) bronchospasm.
- Heparin rebound: reappearance of hypocoagulability after adequate neutralization of heparin, which was measured by the increasing ACT after operation than the final ACT after neutralization in operation.
- Intraoperative blood loss: all the gauzes used for bleeding were placed on the physical balance and weighed, then the original weight was subtracted from these gauzes, and the difference was the weight of blood lost which was converted into milliliters by dividing the weight by specific gravity, which is 1.055.

### Statistics

All statistical analyses were performed using the computer program SPSS 18.0. Results were expressed as mean±standard deviation. An independent 2-sample Student's t test was employed to analyze continuous data. For the association between categorical variables, we used X^2^. We considered a *P* value <0.05 statistically significant.

## RESULTS

All patients' preoperative characteristics are shown in Table 1. Patients' characteristics had no statistical difference between the two groups.

**Table 1:** Baseline and procedural characteristics after matching.

Variables	Group A (n=168)	Group B (n=230)	*P* value
Age (years old)	54.11±19.16	53.97±19.55	0.9433
Male	81 (48.21%)	102 (44.34%)	0.4446
Obesity (BMI >30 kg/m^2^)	75 (44.64%)	89 (38.70%)	0.2338
Smoking	91 (54.17%)	105 (45.65%)	0.0933
NYHA class III-IV	40 (23.81%)	52 (22.61%)	0.7790
Previous myocardial infarction	59 (35.12%)	67 (29.13%)	0.2046
Previous PCI	48 (28.57%)	66 (28.70%)	0.9784
Hypertension	75 (44.64%)	88 (38.26%)	0.2010
Diabetes mellitus	24 (14.29%)	34 (14.78%)	0.8896
Hyperlipemia	99 (58.93%)	110 (47.83%)	0.0285
COPD	18 (10.71%)	23 (10.00%)	0.8169
Prior cerebrovascular accident	8 (4.76%)	11 (4.78%)	0.9924
Segmental cardiac wall-motion abnormalities	78 (46.43%)	84 (36.52%)	0.0469
Extent of CAD			
Left main stem disease	35 (20.83%)	43 (18.70%)	0.5957
3 vessels	86 (51.19%)	107 (46.52%)	0.3573
2 vessels	70 (41.67%)	103 (44.78%)	0.5357
Logistic EuroSCORE	7.6±2.8	7.7±2.5	0.7082

BMI=body mass index; CAD=coronary artery disease; COPD=chronic obstructive pulmonary disease; NYHA=New York Heart Association; PCI=percutaneous coronary intervention

All patients' perioperative characteristics are shown in Table 2. Patients' liver function, renal function, coagulation function, and homeostasis had no statistical difference between the two groups.

**Table 2:** Preoperative characteristics after matching.

Variables	Group A (n=168)	Group B (n=230)	*P* value
**Before operation**			
HB (mg/L)	121.78±16.79	122.13±17.01	0.8386
Creatinine (umol/L)	72.14±11.78	72.42±11.98	0.8167
Glutamic pyruvic transaminase (U/L)	35.98±17.24	36.04±17.87	0.9732
Total bilirubin (umol/L)	15.42±6.67	15.59±6.71	0.8025
pH (blood gas)	7.39±0.08	7.40±0.07	0.1860
Activated partial thromboplastin time (s)	11.21±2.45	11.20±2.46	0.9680
INR	0.98±0.11	0.99±0.10	0.3455
Partial thrombin time (s)	34.78±8.75	35.01±8.87	0.7973
Thrombin time (s)	14.87±3.78	14.79±3.81	0.8357
Fibrinogen (g/L)	3.74±0.98	3.77±1.01	0.7671
Thromboelastography			
Reaction time to clot initiation (s)	3.63±0.77	3.67±0.71	0.5926
Clot formation time (s)	1.33±0.13	1.32±0.12	0.4285
Alpha	73.76±6.76	74.16±6.53	0.5524
Maximum amplitude	65.68±4.44	64.98±4.29	0.1140
**After CPB**			
HB (mg/L)	100.98±20.17	100.76±20.87	0.9162
pH (blood gas)	7.44±0.08	7.43±0.07	0.1860
Activated partial thromboplastin time (s)	11.54±2.75	11.51±2.71	0.9137
INR	0.99±0.22	0.98±0.37	0.7550
Partial thrombin time (s)	34.70±8.95	34.71±8.55	0.9910
Thrombin time (s)	14.89±3.57	14.88±3.67	0.9783
Fibrinogen (g/L)	3.73±0.87	3.74±0.89	0.9111
Creatinine (umol/L)	72.34±12.07	72.79±11.98	0.7124
Glutamic pyruvic transaminase (umol/L)	35.71±16.74	36.14±16.82	0.8009
Total bilirubin (umol/L)	16.71±6.21	16.99±7.01	0.6800
Thromboelastography			
Reaction time to clot initiation (s)	3.59±0.53	3.66±0.69	0.2724
Clot formation time (s)	1.29±0.18	1.31±0.12	0.1847
Alpha	73.90±6.59	74.10±6.66	0.7665
Maximum amplitude	72.12±6.33	72.32±6.43	0.7579

CPB=cardiopulmonary bypass; HB=hemoglobin; INR=international normalized ratio

### Intraoperative Outcomes

Intraoperative data are shown in Table 3. There was no statistical difference between the two groups including preoperative ACT, CPB time, cross-clamp time, number of distal anastomosis, ACT after CPB, cases who need additional protamine, and the additional quantity of protamine (*P*>0.05). However, the last ACT in the operation room, hemostatic time, intraoperative blood loss, and erythrocyte suspension presented statistical difference between the two groups (*P*<0.05).

**Table 3:** Intraoperative data.

Variables	Group A (n=168)	Group B (n=230)	*P* value
Preoperative ACT	209.22±19.91	209.19±20.02	0.9882
Time of CPB (min)	39.23±5.21	39.91±5.13	0.1952
Cross-clamp time (min)	34.12±11.56	34.20±11.73	0.9461
Temperature during CPB (ºC)	28-30	28-30	__
No. distal anastomosis	2.46±0.82	2.50±0.81	0.6286
ACT after CPB	475.27±21.11	477.19±21.20	0.3719
Cases needing additional protamine	117 (69.43)	159 (69.13)	0.9128
Additional protamine (mg)	20.76±4.98	20.34±5.01	0.4081
Last ACT in operation room	182.77±11.42	215.34±14.38	<0.0001*
Hemostatic time (min)	34.19±15.43	49.21±21.84	<0.0001*
Intraoperative blood loss (ml)	635.45±67.52	863.79±110.72	<0.0001*
Erythrocyte suspension (U)	0.54±0.35	1.21±0.55	<0.0001*
Temperature in operation room (ºC)	21-24	21-24	__

ACT=activated coagulation time; CPB=cardiopulmonary bypass

* =*P*<0.05.

The ACT in the first ICU hour presented statistical difference between the two groups (*P*>0.05). Despite neutralizing the ACT to baseline level after surgery, there was a reappearance of anticoagulant activity indicative of heparin rebound as demonstrated by ACT higher than the previous. So, the number of patients requiring small amounts of protamine to control excessive bleeding was higher in the Group B, which has statistical significance (*P*>0.05). Most patients received a small dose (25 mg) of protamine. Blood in tube in the first 12 hours, blood transfusion in ICU, and reinfused blood from cell-saving devices in the first 12 hours also presented statistical significance in the two groups (*P*>0.05) (Table 4).

**Table 4:** Postoperative data in ICU.

Variables	Group A (n=168)	Group B (n=230)	*P* value
ACT in the first ICU hour	198.23±9.38	230.13±10.21	<0.0001*
Temperature in ICU (ºC)	26	26	__
Heparin rebound	126 (75%)	171 (74.5%)	0.8826
Additional protamine (mg)	__	10.33±12.34	<0.001*
Adverse events of protamine	__	__	__
Bleed in tube in first 12 hours (ml)	159.34±20.32	203.32±23.14	<0.0001*
Blood transfusion in ICU (U)	__	0.83±0.99	<0.0001*
Reinfused blood from cell-saving devices in first 12 hours (ml)	__	143.43±23.33	<0.0001*

ACT=activated coagulation time; ICU=intensive care unit

* = *P*<0.05.

### Postoperative Outcomes

Postoperative outcomes are shown in Table 5. No important differences were detected in rate of reoperation, myocardial infarction, or mortality between the two groups.

**Table 5:** Postoperative outcomes.

Variables	Group A (n=168)	Group B (n=230)	*P* value
Surgical mortality	1 (0.60%)	1 (0.43%)	0.8231
Postoperative cardiac dysfunction	1 (0.60%)	1 (0.43%)	0.8231
Re-sternotomy for bleeding	1 (0.60%)	1 (0.43%)	0.8231
ICU stay (day)	2.98±0.95	3.12±1.14	0.1956
Hospital stay (day)	9.92±1.52	10.12±1.41	0.1771
Ventricular arrhythmia	2 (1.19%)	3 (1.30%)	0.9198
Low output syndrome	__	1 (0.43%)	0.3944
Stroke	1 (0.60%)	1 (0.43%)	0.8231
Myocardial infarction	__	__	__
Atrial fibrillation	68 (40.48%)	98 (42.61%)	0.6700
IABP support	3 (1.79%)	4 (1.74%)	0.9721
AKI requiring dialysis	1 (0.60%)	1 (0.43%)	0.8231
Respiratory failure	__	__	__
Pneumonia	2 (1.19%)	3 (1.30%)	0.9251
DSWI	2 (1.19%)	3 (1.30%)	0.9251

AKI=acute kidney injury; DSWI=deep sternal wound infection; IABP=intra-aortic balloon pump; ICU=intensive care unit

### ACT Continuous Change

**Fig. 1:**
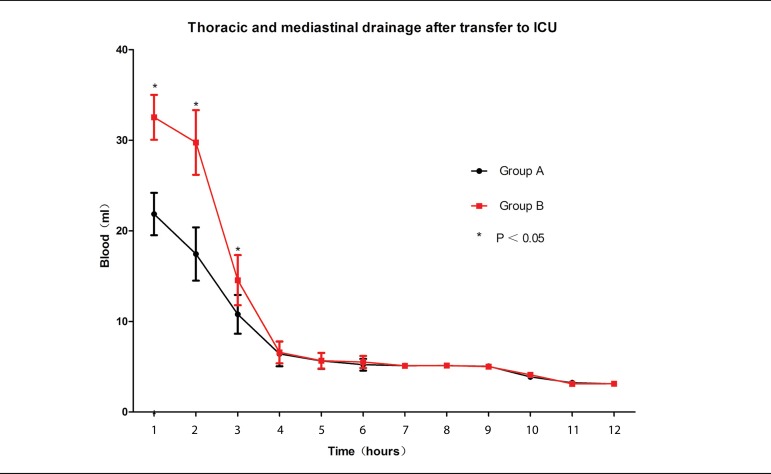
Activated coagulation time (ACT) continuous monitoring. CPB=cardiopulmonary bypass; HTK=histidine-tryptophan-ketoglutarate; ICU=intensive care unit

ACT continuous monitoring is shown in Figure 1. There is no difference until the last neutralization.

### Blood Loss

**Fig. 2:**
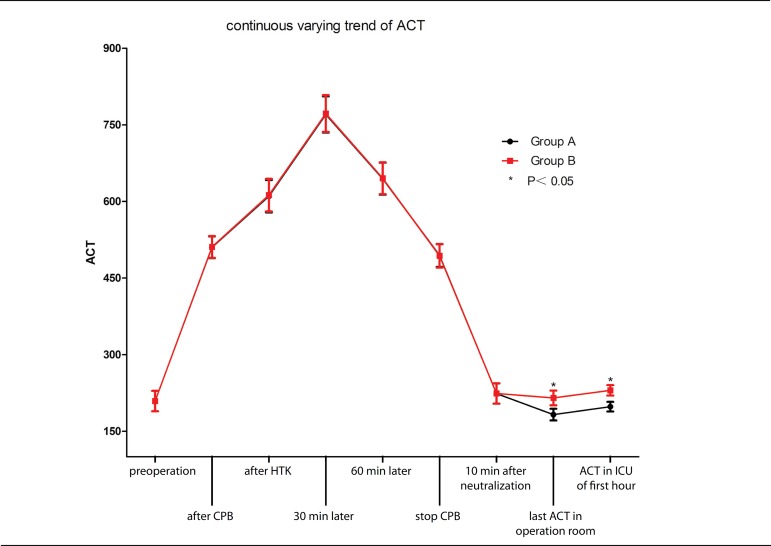
The lower activated coagulation time (ACT) has an effect on reducing mediastinal blood loss. ICU=intensive care unit

The lower ACT has an effect on reducing mediastinal blood loss. As shown in Figure 2, the hourly blood loss in the low ACT group was generally lower than in the Group B in the first 3 hours, which has significant difference (*P*<0.05). However, there was no difference after 3 hours between the two groups.

## DISCUSSION

Protamine has been routinely administered after CPB in order to neutralize the effects of heparin for a long time. The dose of protamine used for neutralization was verified by the ACT value, which must be within normal parameters (100-140 seconds) or at the basal pre-heparin value observed in any case. Many studies aimed to evaluate the dose of protamine for neutralization^[2,3]^ which can contribute to coagulopathy if there is an excess^[4]^ or deficit. However, the protamine dose for neutralizing the heparin effects was influenced by multiple factors, such as hypothermia, hemodilution, homeostasis, and so on^[5,6]^; because of that, a dosage cannot be determined, so most centers used a dose of protamine equal to 1 or 1.5 times the initial dose of heparin and the final ACT was returned to the basal value or a little higher than the pre-heparin value.

However, we found out that after administering protamine (1:1 heparin) and adding additional protamine according to the ACT value after the first neutralization, some cases presented ACT values a little lower than pre-heparin ACT before transfer to ICU. According to the anesthesiologists' experience, because of ACT value is always higher than pre-heparin ACT after first neutralization, 10-25 mg of additional protamine would be administered. So, after 20-30 minutes, the ACT value would be lower than pre-heparin ACT in some cases. Still, the closing time was quicker than of patients with final ACT longer or equal than pre-heparin ACT. In addition, after transfer to ICU, the pericardial and mediastinal drainage in the first 12 hours was also decreasing. So, we retrospectively analyzed cases with final ACT a little lower than pre-heparin ACT and their closing time, bleeding of first 12 hours, and perioperative complications and then compared these situations with cases whose final ACT was longer than or equal to pre-heparin ACT. In our study, we aimed to evaluate the final ACT after the last neutralization, so we can skip many interference factors, such as internal environmental disorder and fluctuation of temperature on coagulation.

In recent years, TEG^[7]^ has become available to measure several aspects related to coagulation. It can show several coagulation aspects, such as blood heparin levels, parameters, and the shortage of PLT. We used TEG to evaluate the PLT function and fibrinolysis situation after neutralization, which was influenced by heparin, protamine^[8,9]^, CPB, and other factors, to exclude the influence of coagulation on bleeding and closing time. In addition, we monitored other parameters, like temperature and blood gas, before measuring the last ACT to exclude other factors that could influence on the closing time and postoperative bleeding. There were no significant differences between the two groups. Also, heparin rebound, hyperfibrinolysis, and an acquired PLT defect were considered to be the main contributors to postoperative bleeding.

Heparin rebound^[10]^ has been identified in many studies. This occurs because a proportion of heparin remains nonspecifically bound to plasma proteins and vascular cells, which can not be cleared by protamine, and that dissociates over time to produce an anticoagulant effect. The incidence of heparin rebound varies widely in the literature and it has been reported to be as high as 50%^[11,12]^. Our study showed that incomplete heparin reversal and heparin rebound were a very common phenomenon after CPB, occurring in 75% of patients in Group A and 74.5% of patients in Group B. Heparin rebound would cause ACT rising directly, and according to this rising ACT, an additional small dose of protamine (25 mg) was used in Group B; but no protamine was used in Group A because the rising ACT was at the same level as the pre-heparin ACT. Although the different side effects of the administration of an additional small dose of protamine, such as anaphylactic reaction, hypotension, and pulmonary hypertension, were not found in Group B, in the Group A, these sides effects were avoided by low ACT before returning to ICU which was safer than the Group B.

Cardiopulmonary surgery is an inflammatory condition that can stimulate the synthesis of acute phase proteins that could contribute to increased protein binding. In addition, prolonged blood contact with the artificial surface of CPB will also affect PLT function which influences on the postoperative bleeding. Surgical trauma, high doses of heparin, and hypothermia all induce the activation of the inflammatory, coagulation, and fibrinolytic systems and the PLT dysfunction, leading to postoperative coagulopathy. After comparing all TEG parameters, preoperatively and after the last neutralization, no significant difference was found between the two groups and the PLT function was normal, probably due to the short CPB time. The PLT count is normal in both groups with no significant difference on the routine blood test.

The authors evaluated closing time, postoperative bleeding, and need for blood product transfusions. The time of operation was shorter in the Group A due to the quickly closing time than in the Group B. The hourly blood loss reduction between the 2 groups was modest (15-30 mL per hour on average), but there was a significant difference in the first 3 postoperative hours. There was no difference after 4 hours, because of the additional protamine in the Group B after the finding of more drainage. We just analyzed the first 12 hours drainage because after this period, drugs would be administered to some of the patients, which would interfere on coagulation factors. There were more blood product transfusions in the Group B than in Group A because of more drainage.

So, why Group A patients with lower final ACT than pre-heparin ACT had positive effect on reducing closing time and postoperative bleeding? Firstly, ACT of all patients was longer than normal preoperative ACT because low molecular weight heparin (LMWH) as anticoagulant was administered 2 times a day until the operation day. The ACT of all patients was longer than normal and always up to 200-220 before operation. Thus, even with neutralization lower than pre-heparin, the final ACT in Group A before leaving the operation room was also longer than normal. The reversion of the heparin effects after CPB referring to abnormal preoperative ACT was not useful for these patients in need of anticoagulation preoperative therapy. If the protamine has neutralized all heparin in the blood, the additional protamine was used to neutralize LMWH (60%), which made the ACT lower than the preoperative ACT. Secondly, heparin rebound was found in almost all patients, which would cause ACT longer after transfer to ICU. So, in Group A, the final ACT was lower than pre-heparin ACT after neutralization and remained equal or a little higher than pre-heparin ACT after heparin rebound, which did not require the use of additional protamine to neutralize it. But, in the Group B, ACT rose much higher than preoperative ACT after heparin rebound, so additional protamine had to be used for neutralizing it to prevent bleeding.

Some people may say that the final ACT lower than the pre-heparin ACT after neutralization may suggest the excessive use of protamine. It is also well documented that the excessive protamine leads to prolonged ACT, weakened clot structure, altered clot kinetics, and PLT dysfunction with subsequent anticoagulant action^[13]^, especially if more than 1.3 mg of protamine, per each milligram of heparin, is administrated. So, we used TEG to evaluate the fibrinolytic systems and PLT dysfunction between the two groups after the final neutralization and there were no differences in all TEG parameters.

## CONCLUSION

In summary, we have demonstrated that lower final ACT after neutralization is safe and has the advantage of leading to lower operative times, bleeding, and post-operative transfusions.

### Limitation

This study has several limitations. Firstly, it is a retrospective, observational, single-centre study, which may influence on the generalizability. So, a final determination would need a prospective, multi-centre study with larger sample size. Secondly, ACT does not correlate with true heparin levels, so it is not accurate to infer the rising ACT caused by heparin rebound. Thirdly, some patients got fever after operation, which will influence on the coagulation system. Finally, we do not know the effect of LMWH on final ACT, because we did not monitor the plasma concentration of LMWH, which remained in the blood and it was not cleared.

**Table:** 

**Authors’ roles & responsibilities**	
WW	Conception and design of the work; acquisition of data; analysis and interpretation of data; drafting the paper; revising the work; approval of the final version
YW	Conception and design of the work; acquisition of data; analysis and interpretation of data; drafting the paper; revising the work; approval of the final version
JW	Conception and design of the work; acquisition of data; analysis and interpretation of data; drafting the paper; revising the work; approval of the final version
RX	Conception and design of the work; acquisition of data; analysis and interpretation of data; drafting the paper; revising the work; approval of the final version
JC	Conception and design of the work; acquisition of data; analysis and interpretation of data; drafting the paper; revising the work; approval of the final version
WZ	Conception and design of the work; acquisition of data; analysis and interpretation of data; drafting the paper; revising the work; approval of the final version
HC	Conception and design of the work; acquisition of data; analysis and interpretation of data; drafting the paper; revising the work; approval of the final version
FX	Conception and design of the work; acquisition of data; analysis and interpretation of data; drafting the paper; revising the work; approval of the final version
XK	Conception and design of the work; acquisition of data; analysis and interpretation of data; drafting the paper; revising the work; approval of the final version
XK	Conception and design of the work; acquisition of data; analysis and interpretation of data; drafting the paper; revising the work; approval of the final version
